# Correlation of Gastroduodenoscopic Findings With Histopathological Diagnosis of Gastroduodenal Biopsy

**DOI:** 10.7759/cureus.84902

**Published:** 2025-05-27

**Authors:** Mahima Choudhary, Sunita Yadav, Rajesh Kumar, Krishna Yadav, Mili Sengar, Rishabh Chaudhary

**Affiliations:** 1 Pathology, Hind Institute of Medical Sciences, Barabanki, IND; 2 Microbiology, Hind Institute of Medical Sciences, Barabanki, IND; 3 Pediatrics, Dr. Ram Manohar Lohia Institute of Medical Sciences, Lucknow, IND; 4 Community Medicine, T.S. Misra Medical College, Lucknow, IND

**Keywords:** duodenal biopsy, endoscopy, gastric biopsy, gastroduodenoscopy, histopathology examination

## Abstract

Background

Endoscopic biopsies, which are performed for the diagnosis of gastric and duodenal lesions, are not pathognomonic and require histopathological confirmation. The aim of the present study was to determine the morphological spectrum of gastroduodenal lesions on histology and to find out the association between endoscopic and histopathological diagnosis.

Methods

This was a prospective observational study carried out at the department of pathology of a tertiary-care teaching hospital in northern India. Clinical details and endoscopic findings of all subjects were noted. The biopsies were obtained in 10% formalin and processed as per standard paraffin embedding techniques. Written informed consent from the participant and approval from the institutional ethics committee were obtained. SPSS software version 24 (IBM Corp., Armonk, USA) was used for the analysis of results. Pearson's chi-square tests and Fisher's exact two-tailed test were used to compare the category variables. Statistical significance was defined as a p-value < 0.05.

Results

A total of 102 gastroduodenoscopic biopsies were assessed. Fifty-seven were gastric and 45 were duodenal biopsies. In gastric biopsies, out of 39 benign and eight malignant lesions diagnosed by endoscopy, 25 were confirmed by histopathology as benign and seven as malignant. In duodenal biopsies, out of 17 benign lesions and four malignant lesions diagnosed by endoscopy, 14 were confirmed by histopathology as benign and three as malignant.

Conclusion

Although we can rely on the gastroduodenoscopy to an acceptable extent in diagnosing malignant and benign lesions of the gastric and duodenal lesions, endoscopic observations alone are insufficient for definitive diagnosis of most of the lesions. The study suggests that all the findings of endoscopy should be combined with histopathological analysis to diagnose the gastric and duodenal lesions accurately.

## Introduction

The gastrointestinal tract (GIT) is the common site of numerous lesions, like congenital, inflammatory, and neoplastic conditions [1]. Endoscopy and histopathology are complementary to each other for facilitating the diagnosis of different lesions in the GIT [2]. Endoscopic biopsies are performed for diagnosis of the disease, determining its extent and responses to therapy, monitoring the course of disease, and for early detection of complications [1]. The stomach and duodenum are frequent sites for malignancies. Stomach carcinoma is the second most common malignancy worldwide [3, 4]. According to the National Cancer Registry of India, neoplasms of the gastric and duodenal regions are the most frequently diagnosed malignancy in males in India [5]. Early detection of malignancy significantly increases patients' survival rate. Early gastric cancer's 5-year survival rate is more than 90% [6]. Endoscopy detects gastroduodenal lesions like atrophy, intestinal metaplasia, and dysplasia at an early stage and prevents their progression to malignancy. The gastroduodenoscopy is the widely accepted diagnostic modality for the diagnosis and treatment of numerous gastric and duodenal lesions [7]. However, it has been perceived that endoscopic findings are not pathognomonic, and they require histopathological confirmation. There is a dearth of studies correlating gastroduodenoscopic findings with gastroduodenal biopsy in Northern India. The aim of the present study was to determine the morphological spectrum of gastroduodenal lesions on histology and to find out the association between endoscopic and histopathological diagnoses.

## Materials and methods

Study design

This was a prospective observational study carried out at the department of pathology in collaboration with the department of gastroenterology and medicine of a tertiary-care teaching hospital in northern India. The study was conducted between February 2022 and January 2024. The study population was patients who had undergone gastroduodenoscopy and gastroduodenal biopsy for various chronic gastrointestinal symptoms like abdominal pain, dyspepsia, recurrent vomiting, etc. Sample size was calculated by SPSS version 24 software (IBM Corp., Armonk, USA) considering the power of the study (80%) and the two-sided significance level (0.05). Exclusion criteria were a previous definitive diagnosis of GIT problems, incomplete clinical details, and insufficient and autolyzed specimens. The histopathological diagnosis was taken as the gold standard for final diagnosis in the present study. All relevant clinical details, including age, sex, clinical findings, and endoscopic findings of these patients, were noted. Prior to enrollment in the study, written informed consent was obtained from all study participants. The study protocol was approved by the Institutional Human Ethics Committee of Hind Institute of Medical Sciences (HIMSAHEC/2023-24/Dr. Mahima Choudhary).

Endoscopy and laboratory work

Endoscopy of the gastroduodenal system was done by a flexible fiber-optic video endoscope (Olympus GIF, Type-Q 150; Olympus Corporation, Tokyo, Japan). Endoscopic findings were noted during gross visualization of the gastroduodenal region, and a biopsy was taken from the suspicious area with the help of biopsy forceps. The tissue biopsies were obtained in 10% formalin, properly labeled, and processed as per standard paraffin embedding techniques. Sections were stained with hematoxylin and eosin (H&E) for the histopathological examination of the biopsy. Giemsa stain was also performed to detect the presence of *Helicobacter pylori*. The diagnosis of gastroduodenal biopsy was made considering the updated revised Sydney system and analyzed with endoscopic findings of patients [8].

Statistical analysis

The statistical analyses were done by SPSS software version 24. The categorical variables were compared with Pearson’s chi-square test and Fisher’s exact two-tailed test, and a p-value < 0.05 was considered statistically significant. Degrees of freedom and effect sizes were also calculated.

## Results

A total of 102 gastroduodenoscopic biopsies were assessed. Out of these 102 biopsies, 57 were gastric and 45 were duodenal biopsies. The most frequent age group in the present study was 31-40 years, with ages ranging from 14 to 78 years and a mean age of 43.37 ± 15.66 years. The majority of gastric biopsies are obtained from patients above 50 years old, and the majority of duodenal biopsies are obtained from those above 30 years old. The male-to-female ratio was 2.5:1. The most frequent complaints in the patients of the present study were dyspepsia (40/102, 39.2%), followed by pain in the abdomen (31/102, 30.4%) and recurrent vomiting (29/102, 28.4%). Patients were also presented with complaints of chronic diarrhea (17/102, 16.7%), anemia, jaundice, and hematemesis (4/102, 3.9%). Out of 102 biopsies, the majority of lesions were in the antrum (37/102, 36.3%), followed by the fundus (12/102, 11.8%) in gastric lesions, and the D2 site (31/102, 30.4%) comprised the majority of the lesions in the duodenum.

Endoscopic findings of gastric and duodenal lesions

In the present study, the endoscopic impression of gastric lesions was mostly benign (39/57, 68.4%), followed by suspicious for malignancy (9/57, 15.8%), malignant (8/57, 14.0%), and normal (1/57, 1.8%) (Table 1). The endoscopic findings in the gastric region showed maximum cases of ulcer (18/57, 31.6%), followed by erosive gastropathy (12/57, 21.1%). The endoscopic impressions in the duodenal region were mostly benign (17/45, 37.8%), followed by normal (16/45, 35.6%), suspicious for malignancy (8/45, 17.8%), and malignant (4/45, 8.9%) lesions (Table 2). The endoscopic findings in the duodenal region comprised mostly normal mucosa (16/45, 35.6%), followed by gastric outlet obstruction (6/45, 13.3%).

**Table 1:** Distribution of Gastric Lesions on the Basis of Endoscopic Findings and Histopathology

Nature of Gastric Lesion	Endoscopic Findings		Histopathological Findings	
	N	%	N	%
Normal	1	1.8	9	15.8
Benign	39	68.4	29	50.9
Suspicious for malignancy	9	15.8	8	14.0
Malignant	8	14.0	11	19.3
Total	57	100	57	100

**Table 2:** Distribution of Duodenal Lesions on the Basis of Endoscopic Findings and Histopathology

Nature of Duodenal Lesion	Endoscopic Findings		Histopathological Findings	
	N	%	N	%
Normal	16	35.6	11	24.4
Benign	17	37.8	31	68.9
Suspicious for malignancy	8	17.8	0	0.0
Malignant	4	8.9	3	6.7
Total	45	100	45	100

Histopathological diagnosis of gastric and duodenal lesions

**Figure 1:**
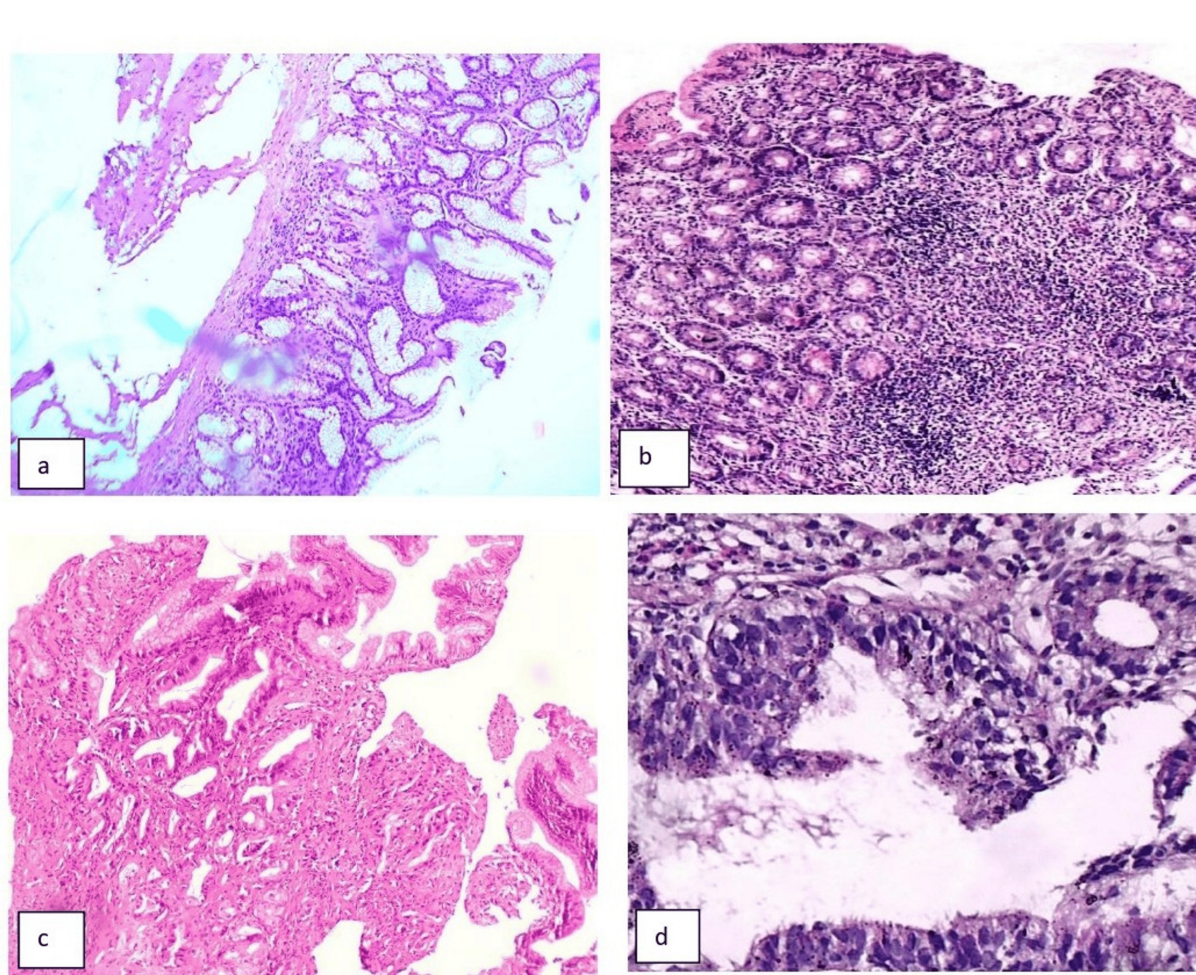
Photomicrograph of Gastric and Duodenal lesions (a) Chronic gastritis with *Helicobacter pylori*; (b) chronic duodenitis with villous shortening; (c) well-differentiated adenocarcinoma of the stomach; (d) well-differentiated adenocarcinoma of the duodenum -- all stained with H&E and viewed at 400x magnification.

The histopathological impression of the 57 gastric biopsies was mostly benign (29/57, 50.9%), followed by malignant (11/57, 19.3%), normal (9/57, 15.8%), and premalignant (8/57, 14.0%) cases (Table 1). The histopathological findings of the gastric biopsies were mostly chronic gastritis (18/57, 31.6%), followed by no significant pathology (9/57, 15.8%) and adenocarcinoma (11/57, 19.3%) (Figure 1a, 1c). In the present study, the histopathological impression of the 45 duodenal biopsies was mostly benign (31/45, 68.9%), followed by normal (11/45, 24.4%), malignant (3/45, 6.7%), and no premalignant case (Table 2). The histopathological findings of the duodenal biopsies were mostly chronic duodenitis (18/45, 40.0%), followed by no significant pathology (11/45, 24.4%) (Figure 1b). Among the malignant cases, only three well-differentiated types of adenocarcinoma were seen (Figure 1d).

Association between endoscopic findings and histopathological diagnosis of gastric lesions

In the present study, we observed the association of endoscopy and histopathological impression of 57 gastric lesions. Out of 39 benign lesions diagnosed by endoscopy, 25 were confirmed by histopathology as benign, three as malignant, six as normal, and five as pre-malignant. Out of eight malignant lesions diagnosed by endoscopy, seven were confirmed by histopathology as malignant, and one was confirmed as benign. And one normal imaging diagnosed by endoscopy was confirmed as normal histology by histopathological examination. Out of nine suspicious malignant lesions diagnosed by endoscopy, three were confirmed by histopathology as benign, one as malignant, two as normal histology, and three as pre-malignant. There was a good association of endoscopic impression and histopathological impression in gastric lesions (p-value = 0.038) (Table 3).

**Table 3:** Association Between Endoscopic and Histopathological Findings of Gastric Lesions

Histopathological Impression of Gastric lesions	Endoscopic Impression					χ^2 ^(df)	p-value	v(effect size)
	Normal	Benign	Suspicious for Malignancy	Malignancy	Total			
Normal	1	6	2	0	9	8.04(3)	0.038	0.38
Benign	0	25	3	1	29			
Pre-malignant	0	5	3	0	8			
Malignant	0	3	1	7	11			
Total	1	39	9	8	57			

Association between endoscopic findings and histopathological diagnosis of duodenal lesions

In the current study, we observed the association of endoscopy and histopathological impression of 45 duodenal lesions. Out of 17 benign lesions diagnosed by endoscopy, 14 were confirmed by histopathology as benign. Out of four malignant lesions diagnosed by endoscopy, three were confirmed by histopathology as malignant, and one was confirmed as normal histology. Out of 16 normal imaging diagnoses by endoscopy, 11 were confirmed by histopathology as benign, and five had normal histology. Out of eight suspicious malignant lesions diagnosed by endoscopy, six were confirmed by histopathology as benign, and two had normal histology. There was a good association of endoscopy and histopathological impression in duodenal lesions (p-value < 0.017) (Table 4).

**Table 4:** Association Between Endoscopic and Histopathological Findings of Duodenal Lesions

Histopathological Impression of Duodenal lesions	Endoscopic Impression					χ^2 ^(df)	p-value	v(effect size)
	Normal	Benign	Suspicious for Malignancy	Malignancy	Total			
Normal	5	3	2	1	11	10.01(3)	.017	0.47
Benign	11	14	6	0	31			
Malignant	0	0	0	3	3			
Total	16	17	8	4	45			

In patients with abnormal findings on endoscopy, the sensitivity, specificity, positive predictive value (PPV), negative predictive value (NPV), and accuracy for malignant lesions were 83.3%, 35.3%, 47.6%, 75.0%, and 55.2%, respectively, and for benign lesions, 69.6%, 35.3%, 78.0%, 26.1%, and 61.6%, respectively (Table 5). However, for suspicious malignant lesions, endoscopic diagnosis sensitivity, specificity, PPV, NPV, and accuracy were very low.

**Table 5:** Accuracy of Endoscopic Diagnosis in Gastroduodenal Lesions

S. No.		Malignant	Benign
1	Sensitivity	83.3%	69.6%
2	Specificity	35.3%	35.3%
3	Positive Predictive Value	47.6%	78.0%
4	Negative Predictive Value	75.0%	26.1%
5	Accuracy	55.2%	61.6%

## Discussion

In the present study, 102 patients with an age range of 14-78 years participated. It was observed that the majority of the patients with gastroduodenal lesions belonged to the 31-40 years (25.0%) and 51-60 years (18.0%) age groups. The age-related differences may be caused by different kinds of risk factors at different ages. This study was supported by Hirachand et al., in which the 16-84 years age group showed maximum distribution of upper gastrointestinal lesions [5]. Although Somani and Patil observed that gastric biopsies were mostly found in the fifth decade (34.50%) [6]. Similar to the present study, Vijaybhasker et al. found that in gastroduodenal biopsies, the age range was 16-85 years, and the commonest age group was 41-50 years [9].

In the current study, the male:female ratio was observed to be 2.5:1. Duodenal biopsies were taken from 35 (77.7%) males and 10 (22.7%) females, and gastric biopsies were taken from 38 (66.7%) males and 19 (33.3%) females. The male:female ratio in the present study for duodenal biopsies was 3.5:1, and for gastric biopsies was 2:1. There was male preponderance both in duodenal and gastric biopsies in the present study, supported by the study of Rani et al. In their study, they found that the overall male:female ratio was 1.5:1, meanwhile, Islam et al. reported a ratio of 1.4:1 [10,11]. Upper gastrointestinal tract lesions were more common in males as compared to females. This indicates that risk factors affect more males than females. A study by Somani and Patil also showed that male patients were more affected, and the ratio of males:females was 2.92:1 for gastric biopsies and 2.57:1 for duodenal biopsies [6].

In the present study, the main presenting complaints were dyspepsia (40/102, 39.2%), followed by abdominal pain (31/102, 30.4%), recurrent vomiting (29/102, 28.4%), and dysphagia (4/102, 3.9%). Endoscopy was mostly done to evaluate the cause of dyspepsia. Sharma et al. also found that pain in the abdomen was the most common presenting complaint in their study of 130 gastric biopsies [12]. Somani and Patil said that most of the patients who had gastric malignancy presented with abdominal pain and loss of appetite [6].

In the present study, out of a total of 102 gastroduodenoscopy biopsies, 57 (55.9%) biopsies were obtained from the gastric region and 45 (44.1%) biopsies were obtained from the duodenal region. The stomach was found to be the most common site for gastroduodenal lesions, followed by the duodenum. In the present study, among the gastric region, the majority of lesions were in the antrum (37/102, 36.3%), followed by the fundus (12/102, 11.8%), and in duodenal lesions, the D2 site (31/102, 30.4%) comprised majority of the lesions. These findings were similar to the study of Sharma et al., who found that the antrum was the most common site of gastric biopsy, i.e., at 93.1% [12]. Somani and Patil in their study found that most gastric malignancies arise in the antrum and pylorus [6]. Rani et al. found in their study that the stomach was found to be the commonest region for upper gastrointestinal (UGI) tract lesions, followed by the esophagus and the duodenum [10]. Hirachand et al. in their study found that the commonest region for UGI biopsy was the stomach, followed by the esophagus and the duodenum [5]. In their study, they found that gastric malignancies were seen more in the antrum. The present study showed more malignant cases in the antrum, followed by the fundus, body, and pylorus.

The present study showed that endoscopic impression in the gastric region was mostly benign (39/57, 68.4%), followed by suspicious for malignancy (9/57, 15.8%), malignant (8/57, 14.0%), and normal (1/57, 1.8%). The endoscopic findings in the gastric region showed maximum cases of ulcer (18/57, 31.6%), followed by erosive gastropathy (12/57, 21.1%). Somani and Patil reported growth and thickening of mucosa on endoscopy, which were 21 cases confirmed as malignancy on histopathology [6]. The present study showed eight cases as growth on endoscopy, which were mostly malignant on histology. In the current study, the histopathological diagnosis of the gastric biopsies showed maximum cases of chronic gastritis (18/57, 31.6%), followed by no significant pathology (9/57, 15.8%), adenocarcinoma (11/57, 19.3%), reactive gastritis with florid foveolar hyperplasia (3/57, 5.3%) acute gastritis (2/57, 3.5%) and erosive gastropathy (2/57, 3.5%), etc. Rani et al. found that out of 46 biopsies from the stomach, 32 (69.6%) cases were non-neoplastic lesions, whereas 14 (30.5%) cases were neoplastic, and the most common non-neoplastic lesion in their study was *H. pylori *gastritis (19.55%), followed by fundic gland polyp (17.5%) [10]. Among the neoplastic lesions, the most common was signet ring cell adenocarcinoma (15%), followed by tubular carcinoma (11%), and one case each (2.2%) of lymphoma (diffuse large B-cell lymphoma ) and papillary adenocarcinoma was found [12]. In the study of 55 gastric biopsies by Somani NS and Patil, inflammatory and benign lesions were more than malignant lesions [6]. Chronic non-specific gastritis was more commonly found in non-neoplastic lesions and tubular adenocarcinoma (61.90%), followed by signet ring cell carcinoma (19.04%), which were more commonly found in neoplastic lesions [6]. 

In the present study, the endoscopic impression in the duodenal region was mostly benign (17/45, 37.8%), followed by normal (16/45, 35.6%), suspicious for malignancy (8/45, 17.8%), and malignant (4/45, 8.9%) lesions. The endoscopic findings in the duodenal region comprised mostly normal mucosa (16/45, 35.6%), followed by gastric outlet obstruction (6/45, 13.3%). In the current study, it was observed that on histopathology, 31 (68.9%) cases were benign, followed by 11 (24.4%) normal, three (6.7%) malignant, and no premalignant cases. The majority of the lesions were benign in nature. The histopathological diagnosis of the duodenal biopsies was mostly chronic duodenitis (18/45, 40.0%), followed by no significant pathology (11/45, 24.4%), mild chronic duodenitis (4/45, 8.9%), and adenocarcinoma-well differentiated (3/45, 6.7%), etc. Among the malignant cases, only three well-differentiated type of adenocarcinoma were seen. Rani et al. reported that out of 24 duodenal biopsies in their study, 20 (83.4%) were diagnosed as non-neoplastic and four (16.6%) as neoplastic [10]. Among the non-neoplastic lesions, celiac disease was more common (58.5%), followed by duodenitis (12.5%). Among neoplastic lesions, adenocarcinoma (12.5%) and neuroendocrine tumor G1 (4.1%) were there. Thus, non-neoplastic lesions predominated in duodenal biopsies, and this finding correlated well with the current study. 

In the present study, we observed a statistically significant association of endoscopic and histopathological impressions of gastric lesions (p-value = 0.038). In gastric biopsies, out of 39 benign and eight malignant lesions diagnosed by endoscopy, 25 were confirmed by histopathology as benign and seven as malignant. Out of nine suspicious-for-malignancy lesions diagnosed by endoscopy, three were confirmed by histopathology as benign, one malignant, two normal histology, and three premalignant. This may be due to the endoscopic interpretation of the inflammatory lesion as malignant. The present study was similar to the study conducted by Rani et al.; they also found a good association between endoscopy and histology in malignant gastric cases [10]. Out of 12 growths (suspicious for malignancy) on endoscopy, all were confirmed to be malignant on histopathology (11 carcinomas, one lymphoma). Somani and Patil also showed a good association between endoscopy and histology for malignant cases [6].

In the present study, we also observed a statistically significant association between endoscopic and histopathological impressions of duodenal lesions (p-value = 0.017). Out of 17 benign lesions diagnosed by endoscopy, 14 were confirmed by histopathology as benign. Out of four malignant lesions diagnosed by endoscopy, three were confirmed by histopathology as malignant, and one was confirmed as normal histology. Out of 16 normal imaging diagnosed by endoscopy, 11 were confirmed by histopathology as benign, and five had normal histology. Out of eight suspicious for malignancy diagnosed by endoscopy, six were confirmed by histopathology as benign, and two were normal. This might be due to the endoscopic interpretation of inflammatory lesions as malignant and less experience of the endoscopist. Rani et al. found that among 24 duodenal lesions, there were 13 cases with scalloping of mucosal folds on endoscopy, and all of them were diagnosed as celiac disease on histology [10]. Hence, the endoscopic and histological findings of celiac disease correlated well with each other in their study. Four cases were diagnosed as suspicious of malignancy on endoscopy, out of which three cases were diagnosed as adenocarcinoma of the duodenum on histology, and one case was diagnosed as carcinoid.

In the present study, endoscopic diagnosis of malignant lesions showed 83.3% sensitivity and 35.3% specificity. Benign lesions showed 69.6% sensitivity and 35.3% specificity. Similar to the present study, Eslami et al. found 60% sensitivity and 98.3% specificity of endoscopic diagnosis in patients with cancer [13]. Kato et al. found 76.6% sensitivity and 84.3% specificity of endoscopic diagnosis in their study [14]. The variability in sensitivity and specificity might be contributed to by differences in the experience of the endoscopist.

In patients with abnormal findings on endoscopy, the sensitivity, specificity, positive predictive value (PPV), negative predictive value (NPV) and accuracy for malignant lesions were 83.3%, 35.3%, 47.6%, 75.0% and 55.2%, respectively, and for benign lesions, 69.6%, 35.3%, 78.0%, 26.1% and 61.6%, respectively.

Limitations of the present study were sampling bias, as study subjects were patients who had gastrointestinal symptoms and were referred for endoscopy; therefore, they may not represent the general population and may affect the generalizability of the study. There may be observer variability for the interpretation of endoscopic and histopathological findings, which could affect the accuracy of the results.

In the present study, we observed that endoscopic and histopathological association was achieved in most of the malignant lesions (10/12, 83.3%), followed by benign lesions (39/56, 69.6%). However, 'suspicious for malignant' cases (1/17, 6.67%) did not achieve a significant association. This might be due to the endoscopic interpretation of inflammatory lesions as malignant and less experience of the endoscopist. Thus, the two techniques complement each other in the diagnosis and management of patients with gastric and duodenal lesions. However, the histopathological evaluation is essential for the confirmation of diagnosis. More studies with larger sample sizes will be essential for a better evaluation of the relationship between gastroduodenoscopy and histopathological impression.

## Conclusions

Although we can rely on gastroduodenoscopy to an acceptable extent in diagnosing malignant and benign gastric and duodenal lesions, endoscopic observations alone are insufficient for definitive diagnosis of most lesions. The study suggests that all findings of endoscopy should be combined with histopathological analysis to diagnose gastric and duodenal lesions accurately.
